# The short-term skeleto-dental effects of a new spring for the intrusion of maxillary posterior teeth in open bite patients

**DOI:** 10.1186/s40510-014-0056-7

**Published:** 2014-09-25

**Authors:** Riaan Foot, Oyku Dalci, Carmen Gonzales, Nour Eldin Tarraf, M Ali Darendeliler

**Affiliations:** Discipline of Orthodontics, Faculty of Dentistry, University of Sydney, Sydney, New South Wales 2006 Australia; Sydney Dental Hospital, 2 Chalmers St, Level 2, Surry Hills, NSW 2010 Australia

**Keywords:** Open bite, Molar intrusion, Miniscrew

## Abstract

**Background:**

The technology surrounding temporary skeletal anchorage devices has improved in leaps and bounds. However, no specific auxiliary exists for the intrusion of molars in conjunction with these devices and currently clinicians are forced to make do with available force delivery materials. A new intrusion auxiliary, the Sydney Intrusion Spring (SIS), was designed to facilitate intrusion without frequent need for reactivation or tissue irritation.

**Methods:**

The subjects consisted of 16 adolescent patients (12 females and 4 males) with an average age of 13.1 years (range 12.2 to 14.3 years). All patients were in the permanent dentition with an anterior open bite of ≥2 mm. Four self-drilling miniscrews were placed into the posterior maxillary buccal alveolar bone. The intrusion appliance consisted of a bonded acrylic appliance and the SIS, activated to produce an initial intrusive force of 500 g. Cone beam computed tomograms were taken after miniscrew placement and at the end of active intrusion. Rendered lateral cephalograms were produced and measurements were taken and compared.

**Results:**

All study objectives were achieved in 4.91 months (range 2.5 to 7.75 months). The mean molar intrusion was 2.9 ± 0.8 mm (*P* < .001), resulting in over bite increase of 3.0 ± 1.5 mm (*P* < .001). The intrusion led to a 2.6° ± 1.3° (*P* < .001) clockwise occlusal plane rotation and a 1.2° ± 1.3° (*P* < .01) counter-clockwise rotation of the mandible. Dental measurements showed a significant uprighting and elongation of the incisors. There was no significant extrusion of the lower molars.

**Conclusion:**

The SIS is an effective appliance for the intrusion of maxillary posterior teeth, in conjunction with miniscrews.

## Background

Since the start of the modern era of orthodontics, orthodontists have acknowledged anterior open-bite malocclusion to be one of the most difficult malocclusions to successfully treat and maintain.

Literature describes a wide range of clinical features and cephalometric traits descriptive of skeletal anterior open bite that are common to *most* individuals. However, in any given individual with this type of skeletal dysplasia, several or only a few of the reported characteristics may be observed. A frequent finding however appears to be an inferiorly positioned maxillary process: maxillary posterior vertical excess with concomitant posterior inferiorly tipped palatal plane and an increased posterior and anterior maxillary dentoalveolar height [1,2]. This is thought to be due to the excessive vertical maxillary development.

Several conventional treatment methods have traditionally been proposed to treat these patients. Extra-oral traction like high-pull headgear, either to molar tubes, maxillary splint or functional appliance [3,4], with force magnitudes ranging anywhere from 300 to 1,500 gr and application times ranging from 10 to 24 h per day, have been proposed. Vertical chin cup therapy is another extra-oral approach employed and may also be used alone, with fixed orthodontic appliances or in conjunction with functional appliances [5] to address vertical control of the mandible during skeletal open bite treatment.

Removable functional orthopaedic appliances [6] have also been described in the literature for the treatment of skeletal anterior open-bite malocclusion. Furthermore, passive posterior bite blocks [7], magnetic bite block appliances [8], the multiloop edgewise archwire technique (MEAW) [9], fixed orthodontic appliances in conjunction with anterior vertical elastics, and extraction treatment have all been suggested as viable treatment modalities for this type of malocclusion. These appliances however all had significant drawbacks and/or side effects.

It has also been proposed that 90% of the patients with skeletal anterior open dysplasia are best treated by a combination of orthodontic and orthognathic surgical procedures. Most often the maxilla is the principal focus of surgery in skeletal open bite dysplasia, as the vertical development of the nasomaxillary complex is nearly always excessive in these individuals [10]. Surgical treatment however is highly invasive and costly, has inherent risk involved and cannot be undertaken until vertical growth has been completed.

With the advent of orthodontic temporary skeletal anchorage devices (TSADs), orthodontists for the first time have had a reliable source of noncompliance-based stationary anchorage. Umemori et al. [11] were the first to show the possibility of molar intrusion in humans, using skeletal anchorage. Subsequently, several studies [12-16] have shown maxillary molar intrusion, using skeletal anchorage, to be a viable and reliable treatment modality for skeletal anterior open-bite malocclusion.

The orthodontic auxiliaries used to provide the intrusive force component, however, are crude adaptations of existing auxiliaries, including NiTi coil springs, elastomeric thread, or chain and rubber bands. These auxiliaries were not developed for the purpose of molar intrusion in conjunction with skeletal anchorage devices and therefore, although being adequate at accomplishing the task at hand, all have significant limitations and drawbacks. Elastomeric thread or chain requires frequent replacement and reactivation, whilst NiTi coil springs often cause significant tissue irritation and hyperplasia. No mention could be found in the literature, regarding a specifically designed intrusion auxiliary for use in conjunction with skeletal anchorage devices.

Consequently, a specifically designed spring, the Sydney intrusion spring (SIS), was conceived and developed at the University of Sydney. The SIS is a purposely created intrusion auxiliary, designed for maxillary dentoalveolar buccal segment intrusion, to be used in conjunction with skeletal anchorage devices like miniscrews or miniplates. It is designed to produce a continuous active intrusion force with infrequent need for reactivation, be easy to install, reactivate and remove, and be hygienic with minimal tissue irritation.

For the purposes of this study, the null hypothesis assumed that posterior dentoalveolar intrusion using skeletal anchorage and the SIS provided no statistically significant changes in the cephalometric measurements of the subjects studied. The aim of this prospective study is to evaluate the clinical use as well as the dental and skeletal effects of the SIS.

## Methods

The subjects selected for this study consisted of 16 adolescent patients (12 females and 4 males) with an average age of 13.1 years (range 12.2 to 14.3 years).

Ethical approval was obtained from the Human Research Ethics Committee, SSWAHS No. X10‐0070&HREC/10/RPAH/126. The criteria for inclusion in the study were the following: patients in the permanent dentition with an anterior open bite between the upper and lower incisors of 2 mm or greater; adequate incisor display on smiling and at rest; an increased lower anterior facial height; convex profile; no severe maxillary posterior crowding or rotated teeth; no current habits, such as digit sucking or tongue thrust; no previous orthodontic treatment, trauma and no dental or congenital anomalies; good oral hygiene and no periodontal disease.

The intrusion appliance used for this study consisted of three main parts, the SIS, a bonded acrylic maxillary expander and four miniscrews.

**Figure 1:**
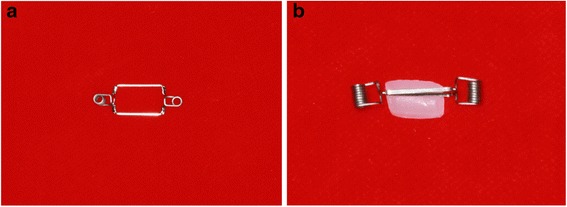
**Th** **e Sydney intrusion spring (SIS).** Side view **(a)** and top view **(b)** .

The SIS consists of two 0.016-in. diameter beta titanium closing loops containing several helices, laser welded to a 0.017 × 0.025 in. diameter beta titanium frame. The closing loops provide low continuous force production over a large range of activation, whilst the frame provides the required stiffness to resist permanent deformation of the spring during activation and placement (Figure 1a,b).

The bonded acrylic appliance consisted of two shallow acrylic bite blocks with an internal wire frame, covering all teeth to be intruded (premolars and molars) and constructed to produce minimal bite opening. The bite blocks were connected by a 7-mm hyrax expansion (Dentaurum, Ispringen, Germany) screw which was bent to allow sufficient palatal clearance for intrusion. The buccal surface of the bite blocks further incorporated two self-ligating brackets (0.022-in. slot, Speed™, Strite Industries, Cambridge, Ontario, Canada) on each side, welded to the internal framework. The brackets were positioned to align with the miniscrews and allow a vertical clearance of 12 mm between the slot on the miniscrew and the slot on the bracket.

**Figure 2:**
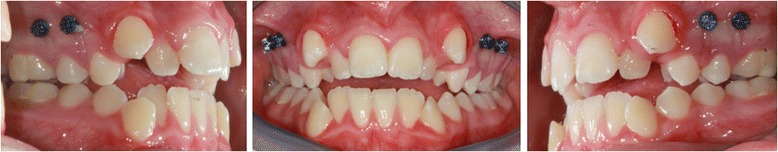
**Miniscrew placement.**

Four self-drilling miniscrews (Aarhus™, Medicon eG, Tuttlingen, Germany; diameter, 1.5 mm; length, 6 mm distributed by American Orthodontics) were placed into the maxillary buccal alveolar bone through the gingiva. The miniscrews were placed between the upper first and second premolar and the second and first molar on both the left and right sides of the maxilla (Figure 2a,b). All miniscrews were placed by a single operator (RF). Alginate impressions were then taken of the upper and lower arches, including the miniscrews.

**Figure 3:**
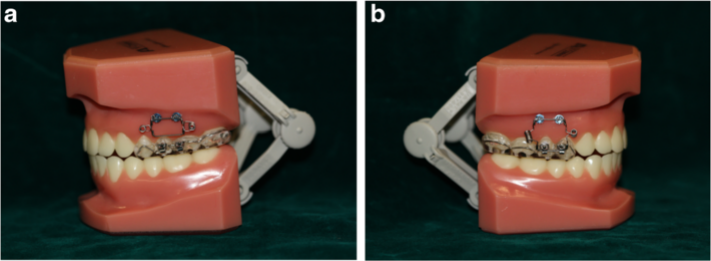
**SIS activation. (a)** Inactive. **(b)** Activated.

After laboratory construction, all bonded appliances were checked for fit and cemented using glass ionomer cement. The SIS was then placed bilaterally and activated to produce an initial intrusive force of approximately 500 g (Figure 3a,b). Loading of the miniscrews was initiated immediately after placement (<48 h) and continued until sufficient intrusion has been achieved. Rapid maxillary expansion was also prescribed, at a rate of 0.25 mm per day, until 30% overexpansion has been achieved. Subjects were seen at 4-week intervals to observe progress, and the springs were reactivated when the force delivery approached 200 g.

**Figure 4:**
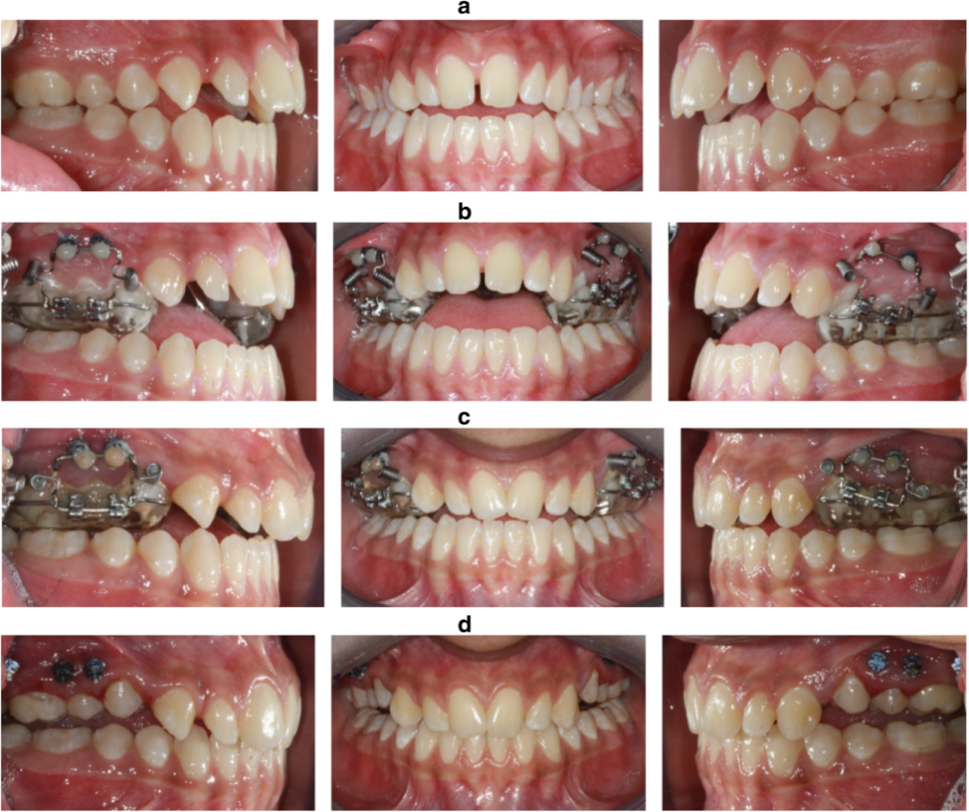
**Achievement of sufficient intrusion. (a)** Initial intra-oral view. **(b)** Intra-oral view at appliance placement. **(c)** Intra-oral view at the end of active intrusion. **(d)** Intra-oral view after appliance removal.

Treatment was continued and sufficient intrusion was deemed to be achieved once the over bite, as measured between the upper and lower incisor tips, minus the bite blocks, reached 2 mm (Figure 4a,b,c,d).

**Figure 5:**
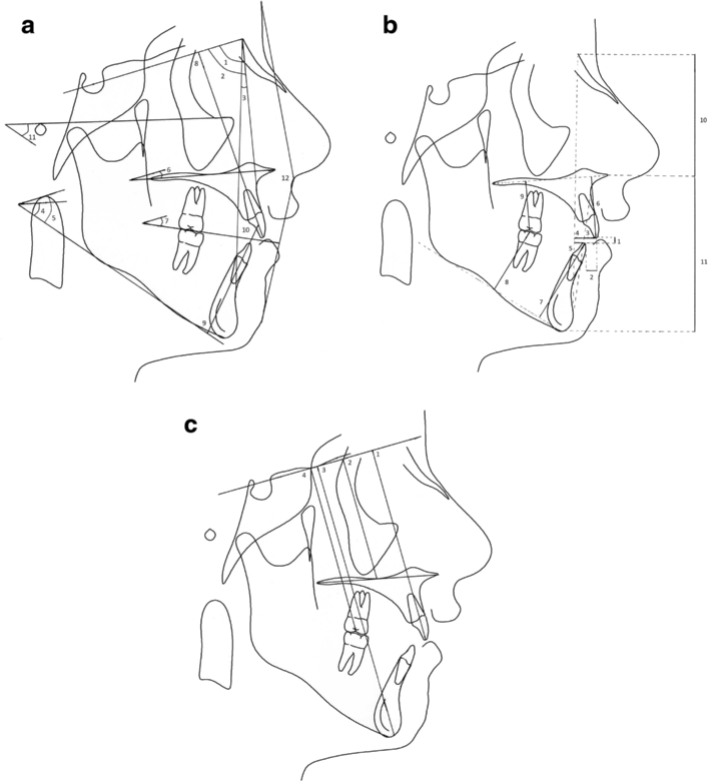
**The cephalometric measurements. (a)** Angular measurements: 1, SNA angle; 2, SNB angle; 3, ANB angle; 4, mandibular plane to palatal plane angle (PP/MP); 5, sella-nasion line to mandibular plane angle (SN/MP); 6, sella-nasion line to palatal plane angle (SN/PP); 7, occlusal plane to sella-nasion line angle (Occ/SN); 8, maxillary central incisor to sella-nasion line angle (U1/SN); 9, mandibular central incisor gonion-gnathion line angle (L1/GoGn); 10, interincisal angle (U1/L1); 11, mandibular plane to Frankfurt horizontal (MP/FH); 12, facial convexity (G′Sn′Po′). **(b)** Linear measurements: 1, over bite; 2, overjet; 3, maxillary central incisor protrusion (U1-Apo); 4, maxillary incisor to nasion-pogonion line (U1-NPo); 5, mandibular central incisor to mandibular plane (L1-MP); 8, mandibular fist molar to mandibular plane (L6-MP); 9, maxillary first molar to palatal plane (U6-PP); 10, upper anterior facial height (UAFH); 11, lower anterior facial height (LAFH). **(c)** SBL measurements: 1, measurement to maxillary central incisor tip (SBL-U1); 2, measurement to palatal plane (SBL-PP); 3, measurement to maxillary first molar occlusal surface (SBL-U6); 4, measurement to gnathion (SBL-Gn).

Cone beam computed tomograms (CBCTs) were taken immediately after miniscrew placement at T1 and at the end of active intrusion, after appliance removal, at T2. The tomograms were acquired using a NewTom 3G (QR, Verona, Italy) with a 12-in. field of view (FOV). The DICOM data obtained from the CBCTs were processed to produce rendered lateral cephalograms, using the Dolphin Imaging system (version 11.0, Dolphin Imaging & Management Systems, Chatsworth, CA, USA). All cephalograms were digitally traced by one investigator (RF) using the Dolphin software. Twenty-three conventional cephalometric measurements were included, consisting of 12 angular and 11 linear measurements [12-14,16] (Figure 5a,b). To negate any possible contribution from growth, the stable basicranial line (SBL) [17] was also utilized in four linear vertical measurements: SBL-U6, SBL-U1, SBL-PP, and SBL-Gn (Figure 5c). A further measurement from U6 to PP was done by transferring the PP from the T1 to T2 radiograph by regional maxillary superimposition.

An error measurement study was done to evaluate the intra-examiner reliability, 1 month after the initial tracings. In this study, the method error did not exceed 0.3 mm for the linear variables and 0.4° for the angular variables.

Statistical analysis of the cephalometric study data was performed using the Statistical Package for the Social Sciences (SPSS, version 17.0, SPSS Inc., Chicago, IL, USA) and was used for descriptive statistical analysis, using paired sample *t*-tests.

## Results

One miniscrew was lost from subject 1, and this subject was subsequently removed from the study. The final subject population therefore consisted of 11 females and 4 males with an average pretreatment open bite of 2.6 mm (range 2.1 to 6 mm). The study objectives were achieved in all patients, and the average intrusion time was 4.91 months (range 2.5 to 7.75 months).

The data from the pretreatment and post intrusion lateral cephalograms, local superimpositions as well as results of the paired *t-*test are summarized in Table 1. Statistically significant changes were observed in several key cephalometric parameters.

**Table 1:** **Su** **m** **ma** **rized cephalometric changes from T1 to T2**

**Cephalometric variables**	**T1**		**T2**		**Difference**		***P*** **value**	**Significance**
	**Mean**	**SD**	**Mean**	**SD**	**Mean**	**SD**		
ANB (°)	4.8	2.9	4.5	2.8	−0.4	0.9	.139	NS
SNA (°)	81.6	5.7	81.8	5.8	0.2	0.5	.183	NS
SNB (°)	76.8	4.8	77.4	5.4	0.6	1.0	.054	NS
PP/MP (°)	31.8	3.8	30.7	4.3	−1.0	1.3	.009	**
U1-SN (°)	107.3	8.5	102.8	8.3	−4.5	3.3	.000	***
Occ plane to SN (°)	17.1	4.4	19.6	4.7	2.6	1.3	.000	***
SN-MP (°)	36.1	4.9	34.9	5.3	−1.2	1.3	.002	**
SN/PP (°)	7.1	2.7	7.0	2.6	−0.1	0.6	.647	NS
L1-GoGn (°)	97.5	8.7	91.0	7.9	−6.6	3.8	.000	***
Interincisal angle (°)	119.5	12.2	131.6	11.6	12.2	5.1	.000	***
(G′Sn′Po′) (°)	160.5	5.5	160.1	5.8	−0.4	1.4	.256	NS
FMA (MP/FH) (°)	27.8	3.5	26.9	3.6	−0.9	1.2	.015	*
Over bite (mm)	−2.2	1.7	0.8	1.1	3.0	1.5	.000	***
Overjet (mm)	4.5	2.3	4.4	1.8	−0.1	1.2	.900	NS
U1-Apo (mm)	8.6	3.1	7.4	3.1	−1.2	0.9	.000	***
U1-NPo (mm)	11.1	4.3	9.6	4.0	−1.5	1.2	.000	***
U1-PP (mm)	25.6	2.3	26.5	2.4	0.9	0.6	.000	***
L1-APo (mm)	4.1	3.3	3.0	3.2	−1.2	0.9	.000	***
L1-MP (mm)	34.7	2.8	36.1	2.7	1.4	0.8	.000	***
L6-MP (mm)	27.9	2.9	28.0	2.9	0.1	0.4	.201	NS
U6-PP (mm)	20.6	2.3	17.7	2.4	−2.9	0.8	.000	***
LAFH (mm)	61.8	4.3	60.9	4.6	−0.9	1.1	.009	**
UAFH (mm)	48.6	3.4	48.6	3.3	0.0	0.7	.908	NS
U6-Mx reference plane (mm)	20.5	2.4	17.8	2.3	−2.7	0.7	.000	***
SBL-U6	69.9	4.6	66.7	4.8	−3.2	0.6	.000	***
SBL-U1	82.3	5.7	83.1	5.6	0.9	0.8	.000	***
SBL-PP	52.0	4.0	52.4	3.8	0.4	0.4	.000	***
SBL-Gn	110.6	7.5	109.6	7.5	−1.0	1.4	.016	*

U1: upper incisor; L1: lower incisor, U6: upper first molar; L6: lower first molar, SBL: stable basicranial line.

*p<0.05, **p<0.01, ***p<0.001.

The main study objective, posterior dental intrusion, was shown to be effectively achieved, as is reflected by the parameters measuring this outcome. The U6-PP decreased by 2.9 ± 0.8 mm (*P* < .001), SBL-U6 reduced by 3.2 ± 0.6 mm (*P* < .001) and local superimposition showed a reduction of 2.7 ± 0.7 mm (*P* < .001) to the maxillary reference line. This translated to an average over bite increase of 3.0 mm ± 1.5 mm (*P* < .001). This observed dental intrusion proceeded at an average rate of 0.59 mm/month.

There was significant uprighting and elongation of the upper and lower incisors. The U1/SN was statistically significantly decreased by 4.5° ± 3.3° (*P* < .001) and the U1-PP measurement increased by 0.9 ± 0.6 mm (*P* < .001). The lower incisors uprighted by 6.6° ± 3.8° (*P* < .001) and showed an average elongation of 1.4 ± 0.8 mm (*P* < .001) to the mandibular plane. These changes led to an average increase in the interincisal angle of 12.2° ± 5.1° (*P* < .001).

The posterior dental intrusion caused a clockwise rotation of the upper occlusal plane, leading to an increase in angulation of Occ Pl/SN of 2.6° ± 1.3° (*P* < .001). The intrusion also caused a counter clockwise autorotation of the mandible. This lead to decrease in SN/MP of 1.2° ± 1.3° (*P* < .01), a decrease of 0.9° ± 1.2° (*P* < .05) in FMA and a 1.01 ± 1.44 mm (*P* < .05) decrease in SBL-Gn. These changes translated to a decrease in lower anterior facial height of 0.9 ± 1.mm (*P* < .01).

There was no significant increase in the measurement of the lower molars to the mandibular plane or SBL to the palatal plane. Midfacial skeletal parameters also remained stable.

## Discussion

The literature suggests an inferiorly positioned maxillary process; maxillary posterior vertical excess, with concomitant posterior, inferiorly tipped palatal plane; and an increased posterior and anterior maxillary dentoalveolar height, to be major and frequent characteristics of individuals with a skeletal open-bite malocclusion [1,2]. The treatment objective for these patients should therefore be intrusion of the maxillary posterior teeth, to address the morphological discrepancies present with this malocclusion and to improve facial aesthetics.

The intrusion of posterior teeth, using skeletal anchorage was first shown to be possible by Southard et al. [18] in mongrel dogs, using osseointegrated implants. With the introduction of miniscrews Ohmae et al. [19] subsequently demonstrated the possibility of using miniscrews for the intrusion of mandibular third premolar teeth in beagle dogs. Following the report on molar intrusion in human subjects by Umemori et al. [11], there has been several studies that have shown molar intrusion, using TSAD, to be a viable treatment modality [12-16].

However, no firm evidence exists in the literature regarding the optimal force levels for maxillary dentoalveolar intrusion. The proponents of extra-oral traction, in the form of headgear, advocate relatively high-force magnitudes ranging anywhere from 300 to 1,500 g of force. Most however use forces in the range of 400 to 600 g. In terms of traditional intra-oral tooth borne mechanics, Burstone [20] suggested 20 g of intrusive force for incisors and 50 g for canines, whilst Proffit [21] advocates 10 to 20 g of continuous intrusive force. Kalra et al. [22] applied 90 g of force to intrude molars in children compared to Melson and Fiorelli [23] who suggested 50 g of force for molar intrusion in adults. With the advent of skeletal anchorage, force systems could be altered and increased without concern for unwanted side effects in the reactive unit and Büchter [24] found miniscrews to remain clinically stable at force levels up to 900 g. Using skeletal anchorage, the suggested intrusive force level for single maxillary molars, from literature, appears to be 100 to 200 g [25]. Kato and Kato however found 100 g of force to be insufficient for *en masse* molar intrusion, but smooth progressive intrusion was achieved when the force levels where increased to 300 g per side [26]. The literature therefore seems to suggest optimal force levels of 200-500 g for *en masse* molar intrusion using skeletal anchorage [12-16].

Consequently the SIS, a purposely designed intrusion auxiliary, was developed to produce clinically significant, efficient *en masse* intrusion of posterior teeth. Laboratory tests suggest that the SIS is able to deliver these suggested force levels of 500 to 200 g in a curvilinear fashion from an activation of 5.5 mm down to 0.8 mm.

The present study is the first to investigate the treatment of skeletal anterior open-bite malocclusion, through molar intrusion with TSADs, in an adolescent sample group. Our results, which indicate an average of 2.9 mm molar intrusion and 3.0 mm increase in overbite, however compares favourably to the results of conventional open bite treatment in adolescents. A major shortcoming of our study is that it does not include an untreated control group. The main aim of this paper was to report on the preliminary findings of a newly designed molar intrusion appliance and future long-term studies should include an untreated control group. The SBL (Figure 5c) was utilised to negate any possible contribution from growth, and a further vertical measurement from U6 to PP was done by transferring the PP from the T1 to T2 radiograph by regional maxillary superimposition as well.

Firouz et al. [27] investigated the effect of high-pull headgear in 12 patients with an anterior open bite of at least 2 mm. The headgear was worn for 12 h per day for 6 months and produced an average of 0.54 mm intrusion of the maxillary molars. Orton et al. [28] demonstrated 0.72 mm of molar intrusion when using a maxillary intrusion splint and vertical pull headgear with 500 g of force per side, for 14 h per day. Recently Abudallatif and Keles [29] described the use of an acrylic cap splint expander and occipital headgear to cause intrusion of the maxillary posterior teeth and a clockwise moment on the maxillary dentoalveolar complex. The headgear was activated to produce a force of 500 g per side and was worn for 14 to 16 h per day for 6 months. Results suggested a clockwise rotation of the maxillary dentition, reduction in mandibular plane angle; maxillary molar intrusion of 2.81 mm and an over bite increase of 3.75 mm.

A study by Kiliaridis et al. [30] comparing passive posterior bite blocks to posterior repelling splints suggest that passive blocks are able to produce a vertical over bite correction of 1.5 to 3.0 mm in younger individuals, but that the main improvement seems to occur in the first weeks, followed by a ‘plateau’ period. No figure was given for the amount of molar intrusion achieved.

Investigation into a fixed magnetic appliance, producing an intrusive force of 1,080 g to the upper and lower posterior dentition, by Kalra et al. [22] found an average of 1.6 mm maxillary molar intrusion, 3.8 mm increase in over bite and a mandibular plane angle decrease of 1.3° after 4 months of treatment. However, a 4 month follow up period saw 1.8 mm of eruption of the maxillary molars. Meral and Yüksel [8] studying the MAD IV appliance, a removable appliance consisting of anterior attracting and posterior repelling magnets, producing a reciprocal force of 300 g, found no significant maxillary molar movement, but 0.75 mm of mandibular molar intrusion with the use of this appliance.

The literature on maxillary molar intrusion using TSADs consists primarily of individual case reports and the patients were all adults with long standing anterior open-bite malocclusion, many of whom had refused conventional combined orthodontic-orthognathic surgical treatment.

Kuroda et al. [14] in their prospective study of 10 adult female subjects (mean age 21.6 years) with an anterior open-bite malocclusion of 5.2 mm on average (±1.8 mm), by means of either SAS miniplates or titanium miniscrews; showed an average of 3.6 mm of molar intrusion, 6.8 mm over bite increase, 3.3° reduction in mandibular plane and no elongation of the incisors. These results were achieved in 7 months of active intrusion.

A subsequent study on molar intrusion by Erverdi et al. [13] evaluated the maxillary posterior dentoalveolar segment intrusion in 11 anterior open bite subjects with a mean age of 19.5 years. For this study the authors used two 9 mm NiTi coil springs on each side, increasing the intrusive force to 400 g per side. Results suggest a mean molar intrusion of 3.6 mm, an over bite increase of 5.1 mm and 3.0° reduction in facial convexity. No significant changes were observed in the vertical or angular incisor position. The average treatment time was 9.6 months.

Xun et al. [16] employed both maxillary palatal and mandibular buccal miniscrews to evaluate the posterior dentoalveolar intrusion in 12 subjects (mean age 18.7 years) with a mean pretreatment open bite of 2.2 mm. Results suggest that the maxillary first molars were intruded on average 1.8 mm, mandibular molars 1.2 mm, mandibular plane angle decreased by 2.3° and the over bite increased by 4.2 mm in 6.8 months. They further report 1.3 mm extrusion of both the upper and lower incisors, 5.0° retroclination of the upper and 1.4° of the lower incisors and a 4.4° increase in the occlusal plane to SN.

Data from the current study suggests intrusion of 2.9 mm at the maxillary first molars, an over bite increase of 3.0 mm, mandibular plane decrease of 1.2° and an occlusal plane increase of 2.6°. These results compare favourably to those found by several other studies [12,15,16] but is less than that found by Erverdi et al. [13] and Kuroda et al. [14] The greater intrusion found by these studies might be explained by the fact that the their subject group started with larger pretreatment open bites, Kuroda et al. [14] mean pretreatment open bite: 5.2 mm, and therefore required significantly greater intrusion than our sample, who had an average pretreatment open bite of 2.6 mm.

The significant elongation and uprighting of the upper and lower incisors, with the upper incisors extruding by 0.9 mm and retroclining by 4.5°, whilst the lower incisors extruded by 1.4 mm and uprighted by 6.6°, are in concurrence with the results found by Erverdi et al. [12] and Xun et al. [16].

The active intrusion time in the present study is comparable to that of Sherwood et al. [15] and Erverdi et al. [12], but with greater dentoalveolar intrusion achieved and significantly shorter than several other studies [13,14,16].

Due to the fact that our subject population consist of growing individuals, the contribution of relative intrusion, through vertical maxillary growth, to the total intrusion achieved could not be disregarded. Therefore, additional measurement using regional superimposition to transfer the maxillary plane was done. Measurement from SBL to the palatal plane was also done to further verify the prior. These parameters indicated that there had been virtually no vertical maxillary growth during the treatment intervention period. Relative intrusion has therefore not significantly contributed to the amount of intrusion achieved, as measured to the palatal plane. This may have attributed to the relatively brief duration of the treatment intervention.

The presence of the SIS was well tolerated by all subjects. Subjects also reported little difficulty in adaption and maintenance of the appliance. Subjective clinical observation suggests that the SIS resulted in little or no tissue irritation and no gingival overgrowth, even in the presence of poor oral hygiene. One possible side effect of intrusion using buccal miniscrews could be the roots of the intruding teeth impinging on the miniscrews after intrusion; however, this was not observed in this study, as the miniscrews were placed with enough clearance in the alveolar bone.

Although several previous studies employed titanium miniplate anchorage, self-drilling orthodontic miniscrews with a diameter of 1.5 and 6 mm in length were used in the current study. We believe that these devices are reliable and effective sources of anchorage for molar intrusion. Furthermore, they offer a simple insertion and explanation technique, are relatively affordable and require very little instrumentation for placement and removal. Compared to miniplates, this treatment generally requires a very atraumatic surgical procedure with greater patient comfort and better postoperative sequelae.

Interestingly, several studies suggest increased failure rates of miniscrews in younger adolescent patients. Others further suggest higher than average failure rates in individuals with a hyperdivergent open bite facial pattern, increased mandibular plane angle, high Frankfurt horizontal plane and low upper gonial angles. These factors have mainly been related to a decreased cortical bone thickness in younger patients and individuals with a hyperdivergent skeletal open bite facial pattern [31,32]. These findings however have not been supported by our current data, with only 1 out of the 64 miniscrews placed during the study, failing.

Molar intrusion using skeletal anchorage is a relatively new treatment modality; hence, very limited literature exists on the stability of the intrusion results achieved. Sugawara et al. [33] were the first to report on treatment stability, suggesting a relapse rate of 30% for mandibular molar intrusion 1 year after treatment.

Baek et al. [34] recently were the first to publish an investigation on the long-term stability of maxillary posterior dentoalveolar segment intrusion using skeletal anchorage devices. Their report describes the post treatment changes in nine adult patients (average age 23.7 years), 3 years after treatment. On average, the maxillary molars were intruded by 2.39 mm and experienced eruption of 0.45 mm during the 3-year follow-up period. This equated to a relapse rate of 22.9%, with authors further reporting that 80% of the total relapse occurred in the first year of retention. In parallel with this, the over bite increased by a mean of 5.56 mm during treatment but only relapsed by 1.2 mm in 3 years.

## Conclusions

The SIS, a purposely designed intrusion auxiliary, is an effective appliance for the intrusion of maxillary posterior teeth used in conjunction with miniscrew TSADs.

The appliance is able to achieve clinically significant intrusion over a large range of activation, with minimal maintenance and requirement for reactivation.

The presence of the appliance is well tolerated by patients and resulted in minimal tissue irritation and no gingival overgrowth.
